# The paradox of corporate sustainability: analyzing the moral landscape of Canadian grocers

**DOI:** 10.3389/fnut.2023.1284377

**Published:** 2024-01-12

**Authors:** Samantha Taylor, Sylvain Charlebois, Tammy Crowell, Bryce Cross

**Affiliations:** ^1^Faculty of Management, Dalhousie University, Halifax, NS, Canada; ^2^Accounting Department, Saint Mary's University, Halifax Regional Municipality, NS, Canada

**Keywords:** food policy, retailing, economic growth, inflation, corporate sustainability, accounting, financial reporting

## Abstract

Food prices have experienced unprecedented increases in recent times. Simultaneously, grocers are facing allegations of capitalizing on inflation to generate unjustifiable profits. Escalating expenses and a lack of transparency have engendered heightened consumer skepticism. This perceived presence of barriers and excessive profitability gives rise to ethical concerns. Our case study delves into the ethical landscape surrounding Canadian grocers, aiming to probe the public’s demand for accountability. To comprehend the factors responsible for the transformation in consumer perception of Canadian grocers in 2022, we conducted an analysis utilizing data from consumers, corporate watchdogs, and industry sources. We extended the paradox perspective on corporate sustainability framework to include a historical aspect to use as our analytical lens. This study sheds light on the alterations in circumstances that have led Canadian consumers to question entire industries and accounting practices that were previously considered unproblematic. As a remedy, we recommend the establishment of a mandatory code of conduct for grocers and an enhancement in the transparency of financial reporting. Paradoxically, corporate profits may continue to grow when societal needs are no longer perceived as being neglected or, even worse, exploited.

## Introduction

Commodity prices are surging at levels unprecedented in decades. Consequently, political analysts are attributing this phenomenon to Canada’s major supermarket chains, alleging their engagement in “Greedflation”—which can be defined as the deliberate exploitation of inflationary trends to generate excessive profits (1). “Greedflation” pertains to the perception that soaring prices are driven by unjustifiable or morally questionable factors. Simultaneously, food inflation has reached historic highs.^1^ All three major Canadian grocers outperformed their average performance, with one grocer having its best year ever (3). Does this mean Canadian grocers are guilty of “Greedflation”?

Some may contend that corporate profits are ethically contentious, as they represent the residual ownership share enabling the accumulation of passive income among the ownership class. Further, Svanberg and Svanberg (4) advocate that “to the degree, business ethics shares the conventional perspective, it will face a problem justifying the crux of business; the bottom line.” As labor is required to produce residual income for the owner class, the passive income for one individual represents the lost wages of another.

In their paper, Svanberg and Svanberg (4) revealed that conventional ethics may deem only the means of conducting business as potentially morally justifiable, while not necessarily extending the same ethical judgment to the profits generated. Nonetheless, it is important to acknowledge that disentangling profits from the business itself may present significant practical challenges. Lee (5) advocates for the implementation of policies aimed at enabling firms to maximize their appropriation of the positive externalities arising from their innovations. This approach is intended to enhance the incentives for prospective innovators. It is plausible to argue that corporate profitability plays a pivotal role in driving innovation, even in critical domains such as food safety.

To illustrate this point, consider a contrasting scenario where market conditions are characterized by reduced income levels and limited resources for investment and profit accumulation. In such circumstances, low and middle-income countries may find themselves grappling with foodborne diseases stemming from the consumption of fresh perishable foods, particularly those sold within informal markets.

The connection between corporate profit and innovation is multifaceted, with implications extending beyond conventional economic metrics. By fostering an environment in which firms can effectively harness the positive externalities of their innovations, policymakers can bolster the impetus for future advancements, which may include critical areas like food safety. Conversely, in markets where profit margins are constrained, there exists a heightened risk of suboptimal outcomes, as exemplified by the prevalence of foodborne illnesses in less affluent regions (6).

The moral landscape is a concept that “addresses the interrelationship between landscapes and moral values and judgments” [(7), 191], and is “a hypothetical space representing human well-being […] where the peaks of this landscape are the heights of prosperity, and the valleys represent human suffering” (8) [as cited in Diller and Nuzzolilli (9), 266]. This paper uses the term “moral landscape” in conjunction with paradox perspective on corporate sustainability framework of Hahn et al. (10) to analyze the peaks and valleys of Canadian grocers and their stakeholders.

Canada employs a mixed-market system primarily characterized by free-market principles. However, the federal government does exercise control over certain services, such as postal and air traffic control. Additionally, the Canadian system exhibits certain features akin to a socialist system, exemplified by the provision of retirement benefits and universal healthcare coverage (11). A free-market system, also known as capitalism, is characterized by the ownership and operation of most businesses by individuals who possess their assets and retain their post-tax profits (*ibid*). However, in the current context of soaring food prices, ethical concerns have emerged regarding the sale of food and the practices of Canadian grocers. There is a lack of consensus regarding an acceptable profit threshold within this sector. In the broader business landscape, corporate leadership remains divided on what constitutes ethical conduct when it comes to earnings management (12).

Three major companies dominate Canadian food distribution to consumers (13). These companies operate diverse businesses that encompass food reporting, apparel, beauty, health (including pharmaceuticals), and other general merchandize, all consolidated within a single category. Present reporting standards allow companies with operations sharing similarities in nature, production, or sales to aggregate their financial data. Consequently, this practice renders it challenging, if not entirely unfeasible, to accurately assess profitability solely based on food sales. In the context of an investor call with financial analysts, one president of a grocery chain attributed their increased margins to higher-margin products such as fragrances. When a member of the media sought validation of the grocer’s statement from an accounting professor, the professor indicated that it was impossible to either confirm or refute the statement using the publicly available information in the financial statements (14). Without greater transparency, it remains challenging to determine whether Canadian grocers have engaged in consumer price gouging.

The escalation in prices and the absence of transparency have contributed to a heightened sense of consumer distrust, with more than two-thirds of Canadians perceiving grocers as capitalizing on inflation to impose exorbitant pricing (15). Food constitutes a fundamental human necessity, with other essential requirements such as warmth and shelter being subject to direct regulation or significant government policy influence in Canada. Various components of the Canadian food distribution system fall under substantial regulatory oversight within the country. The Canadian Health Food Association delineates these regulations through three key legislative acts, namely the Food and Drugs Act, Safe Food for Canadians Act, and Consumer Packaging and Labeling Act (16). These acts ensure Canadians have transparent data (labeling, advertising, and claims) to make informed food decisions.

While certain Canadian products, such as dairy and poultry, are subject to government regulation, including domestic production controls, wherein farmers are able to recover the costs of production (17), Canada does not currently have regulations dictating the prices that grocers charge to consumers (17). Although price ceilings may appear to address immediate concerns, implementing price ceilings on food items could potentially diminish consumers’ access to fresh produce, hinder innovation, and limit investments in research (17). Perhaps greater competition and increased food innovation are necessary to address the issue of food inflation in Canada. This may be a key rationale behind the Competition Bureau Canada’s decision to initiate a study into grocery prices. When addressing this inquiry, the Competition Bureau Canada noted, “There are differing perspectives on the causes of these price increases. Some contend that inflation has escalated the cost of goods for grocers, leading to higher prices. Conversely, others argue that grocers are setting higher prices due to a lack of sufficient competition in the market” (18). The Competition Bureau of Canada has articulated the primary objective of its investigation as the pursuit of a deeper comprehension of pertinent issues. The aim is to identify strategies and measures that Canada can undertake to facilitate the competitive and innovative capacities of emerging enterprises (18).

The investigation into how Canada can assist competition (and thus, manage prices) and food innovation may result from a lack of action by the Competition Bureau Canada to date. In 1998, Loblaws acquired Provigo (19), in 2005, Metro acquired A&P (20), and in 2013, Sobeys acquired Safeway (21). In 2021, Loblaw, Metro, and Sobeys had over $100 billion in annual revenues (22) and were responsible for most food sales in Canada (13). Similar consolidations have faced scrutiny and may not be permitted elsewhere. In the United States, grocer consolidation faces opposition to reducing the likelihood of oligopolistic industries. Kroger (23) was trying to acquire Albertsons for approximately $25 billion and become the second-largest grocer in America (24). United States regulators have pushed back and may require the divestiture of a sizeable number of stores, effectively ensuring a competitive market is maintained, an action that both companies say they are willing to make to complete this proposed deal (25). In Canada, consumers are left to ponder whether grocery retailers are exploiting the unprecedented surge in inflation to impose prices that exceed a justifiable increase. Consumers harbor inquiries and apprehensions but find themselves unable to procure satisfactory responses.

Current research indicates that, in the realm of policy matters, manufacturers tend to be perceived as more accountable than farmers and retailers (26). However, recent record-high inflation and the current rise in consumer distrust on popular and social media outlets have signaled that consumer concern may be shifting toward grocery retailers.

The primary objective of our study is to conduct an ethical analysis of the Canadian grocery industry, with a specific focus on examining the moral dimensions of grocers’ conduct. This research seeks to delve into the growing public demand for greater accountability within the industry and, consequently, aims to put forth actionable recommendations to enhance transparency. Notably, the profitability of the grocery sector has hitherto received limited comprehensive scrutiny, making this investigation particularly significant. The unprecedented rise in food prices has raised ethical questions among Canadians, prompting inquiries into whether it is morally justifiable for grocery retailers to reap substantial financial gains while consumers face the challenges of limited alternatives for meeting their essential needs. This study aims to shed light on these critical ethical concerns.

In our study, we investigate the following research question: how have stakeholders perceived the economic consequences of rising food inflation? To examine the complex interplay between profitability and societal welfare, we adopt a paradoxical approach to corporate sustainability. Our investigation focuses on the Canadian economy as a case study to shed light on the escalating tensions between retailers and consumers. In this pursuit, we employ descriptive, instrumental, and normative dimensions of corporate sustainability (10) to observe and understand the moral landscape of the Canadian grocer oligopoly.

We aim to elucidate the factors responsible for the shift in consumer perception of Canadian grocers in 2022. Our analysis encompasses consumer data, corporate watchdog reports, and industry data, with the goal of proposing solutions to bolster trust and improve social and economic well-being within the Canadian grocery landscape. This research seeks to make the following significant contributions. Firstly, we intend to shed light on the evolving conditions that led Canadian consumers to lose trust in entire industries and accounting practices, which had previously not raised concerns. Secondly, we will explore the availability of information to consumers and the limitations therein.

Finally, we will put forth a recommendation for a mandatory grocer code of conduct, encompassing enhanced financial reporting, and conclude by outlining avenues for future research.

The structure of our paper is as follows: In section 2, we will delve into the conceptual framework, which comprises three key antecedents essential to understanding the moral landscape of Canadian grocers—namely, inflation, consumer trust, and the social aspect of sustainability. This will be followed by an explanation of our case study methodology and the specific factors we examined in section 3. Section 4 will present our findings, with the subsequent discussion taking place in section five. We will conclude by offering recommendations for formal agreements and increased transparency to aid grocers in regaining the trust of Canadians. It is our contention that through reestablishing this trust, grocers can not only enhance their positive social impact but also potentially, paradoxically, bolster their net profits, inflationary context, and consumer trust.

To address the tensions between grocers and consumers, we look to first understand the economic, behavioral, and financial antecedents to this conflict.

### Inflation

Hart (27, p. 8) defines inflation as “a process that raises price levels” and “defining it like this, we can graft on adjectives to describe the intensity or mechanism of particular kinds of inflation.” When discussing the consumer market, Glushchenko (28, p. 69) defines “official inflation” as “the growth of the overall level of consumer prices.” He then listed the limitations of its measurement, including a consumer basket of goods often ignores items only purchased by the wealthy. That inflation based on a basket of consumer goods does not directly reflect a “real” reality. An example presented by Glushchenko was if one in 100 people purchase a television set, then one 100th of a television will be in the basket, and that number will be divided by 12 to represent monthly inflation. That “basket of goods” is referred to as the Consumer Price Index (CPI), and each country has its measurement of CPI.

The CPI basket of goods is not static, as evolving societal norms influence consumer preferences. Items that were once deemed luxurious, such as film cameras, may now constitute typical household expenditures (27). Moreover, inflation metrics have failed to account for the fact that consumer packaged goods have diminished in size, for instance, with some products offering fewer chips or a reduced weight compared to their previous iterations.

To communicate the direction and magnitude of rising inflation, adjectives such as “creeping,” “galloping,” and “hyper” may be used (27). Hart likened the latter term, hyperinflation, to prices rising so fast that the purchasing power used to buy commodities would quickly evaporate off the paper money. When identifying inflationary terms and conditions that may harm society, Temple (29) found that while there is no evidence that moderate inflation can inhibit growth, high inflation, defined as over 100% per year, does inhibit growth. Regardless of the amount of inflation, Shaw (30) suggests it is society’s democratic institutions, not the private sector, who should deal with “national agenda issues” like inflation, pollution, and unemployment.

While inflation can be mitigated through systemic policy decisions, the escalation in prices of specific products imposed by corporations on consumers can significantly influence consumer trust.

### Consumer trust

Behavior often drives action. Sirdeshmukh et al. (31) adopted definition of consumer trust of Moorman et al. (32) as a “willingness to rely on an exchange partner in whom one has confidence.” To understand the different types of exchange partners consumers may rely on in the food industry, we look to the literature. Wu et al. (33) find consumer trust in food and the systems that govern food distribution to be tied to both individual food items (i.e., through packaging labels, certifications, country of origin, and food traceability) and with producers, processors, and retailers providing consumers with food safety. The authors acknowledge that government agencies, third-party institutions, advocacy groups, and the media impact how consumers perceive labeling information and how food operators, including grocer retailers, are viewed by consumers.

Consumer mistrust in the food system can impact consumer purchasing behavior. Nuttavuthisit and Thøgersen (34) found that consumers’ mistrust in the food control system had a significant negative impact on purchasing organic food. Further, Macready et al. (26), in our study, we discovered that consumers’ trust in participants within the food supply chain is contingent upon their perception of the participants’ competence, level of care, and degree of openness. When consumers lack confidence in a participant, such as a grocery retailer, demonstrating care, and transparency in their actions, it results in a corresponding erosion of trust among consumers. Similarly, Rampl et al. (35) found integrity as a driver of consumer trust of food retailers.

It is crucial to examine the connections between consumer perception and consumer trust. Research has demonstrated that the emotional attachment between consumers and firms significantly impacts consumer loyalty and word-of-mouth communication (36). They found that place identity was a predictor of emotional attachment. Therefore, it is reasonable to investigate whether a negative place identity (e.g., associated with a grocery retailer) would diminish a consumer’s emotional attachment and subsequently reduce their purchasing loyalty and positive word-of-mouth behavior.

In numerous countries, such as Canada, utilities undergo strict regulation, accompanied by regulations pertaining to housing affordability and housing choice. Conversely, the food retail sector, for the most part, remains unregulated. During periods of mounting financial stress and diminishing consumer trust, the potential consequences on society become more pronounced as families grapple with the challenge of providing sustenance for their loved ones amidst rising corporate profits, potentially resulting in an increasingly adverse social impact.

### Social impact

Corporate social responsibility (CSR) means going beyond financial performance and shareholder interest (10). Given the link between managing systematic risk, portfolio value, and CSR (37), investors want reliable and transparent disclosures to aid in their investment choices.

International investment stakeholders have called for transparent and reliable reporting that goes beyond financial accounting—they want standards on environmental, social, and governance (ESG). As a result, in late 2021, the International Financial Reporting Standards (IFRS) Board announced a new standards board, the International Sustainability Standards Board (ISSB) (38). The ISSB was created to deliver a global baseline for ESG disclosures to ensure international investment stakeholders have a complement of financial and sustainability measures to assist their decision-making process.

Similar appeals for greater openness among participants in the food chain to furnish pertinent information and enhance transparency are not novel. The need for heightened transparency may arise in response to product crises or deliberately engineered events. For instance, enhanced labeling transparency emerged as a response to Canada’s mad cow disease (39) and the manufactured case of salmonella (40). However, what is relatively new is the extension for the call for transparency beyond personal crises toward overall transparency prior to a specific incident (26). It is at that system level that Bansal (41), as summarized by Hahn et al. (10), defined corporate sustainability as “the intersection of the three principles: environmental integrity, social equity, and economic prosperity.”

Previous research has often framed sustainability as a “business case,” focusing on the analysis and discussion of financial outcomes as the primary metric of success, with environmental, social, and governance (ESG) considerations seen as a “cost” that could potentially have a negative impact on a company’s financial performance (10). In contrast, the paradox perspective of sustainability encourages the consideration of the intrinsic value of multiple metrics beyond their direct and immediate impact on the company’s bottom line (10). Hahn et al. (10) discovered that adopting a paradox perspective and evaluating sustainability in terms of descriptive, instrumental, and normative aspects led to superior contributions to business success.

When examining the three dimensions of the paradox perspective on sustainability, the descriptive dimension serves the purpose of both “describing and explaining” sustainability issues, which aim to “capture organizational phenomena surrounding such tensions” [(10), p. 240]. The instrumental dimension establishes connections between determinants and outcomes, encompassing both short- and long-term consequences. In contrast, the normative dimension encourages corporations to be accountable beyond mere financial returns and shareholder concerns, potentially contributing to a broader discourse on the role of business in sustainable development (10).

When consumers are informed that rising grocery store costs are attributed to inflation, without accompanying empirical evidence to either substantiate or refute the claims made by grocers, it exacerbates tensions among consumers, corporate watchdogs, and the retail grocery industry. The prevailing dominant culture and historical lack of transparency in financial reporting are now being challenged more vigorously than ever before.

There is a social and moral impact of consumers facing increased costs to purchase food and provide their families with nutritious meals. The total annual cost of diet-related chronic disease in the United States exceeds 1 trillion dollars (42). Research published in the *Journal of the American Medical Association* found that in the United States, there are nearly 1,000 deaths per day linked to cardiovascular and diabetes disease, with poor diet contributing factors (43). Food insecurity not only has immediate negative consequences but can also lead to long-lasting health and societal impacts.

In order to address the existing tension between Canadian grocers and consumers, our research endeavors to answer the following research questions: How do stakeholders perceive the economic impact of the rising food inflation?

We approach the examination of the conflict between corporate profits and societal welfare through the lens of the paradox perspective on corporate sustainability. To assess the escalating conflicts between grocers and consumers, as well as the role played by industry watchdogs, we employ the descriptive, instrumental, and normative dimensions of corporate sustainability.

This approach allows us to closely examine and comprehend the ethical landscape within which Canadian grocers operate. The tension between consumers and grocers encompasses economic, behavioral, and financial components, and mitigating this tension may yield positive social outcomes. Consequently, we proceed with our analysis of the ethical framework surrounding Canadian grocers.

## Materials and methods

Pragmatism philosophy guides our research methodology (44). We employ an abductive approach, whereby our case study strategy is operationalized by mixed-methods (44) over a longitudinal time horizon and focused on the grocery industry to investigate the evolving consumer perception and trustworthiness of Canadian grocers. Canada’s food retail sector is primarily dominated by five major grocery retailers, three of which are Canadian public companies collectively generating an annual revenue exceeding 100 billion dollars. In our study, we will specifically examine the Canadian public company grocer retailers, namely Loblaw Companies Limited (referred to as Loblaw), Empire Company Limited (the exclusive owner of Sobeys Inc., referred to as Empire/Sobeys), and Metro Inc. (referred to as Metro), which are the three largest Canadian public grocers.

We utilized various techniques and procedures for our case study research strategy while collecting our data and performing analysis (45). When using a case study strategy, Yin (45), as cited by Eckhardt and Dobscha (46), recommends collecting data that includes annual reports, popular media and press releases, and observations of corporate leadership through published interviews. Additionally, we used Interim financial reports to examine current information.

We conducted a comprehensive review of communication from corporate watchdogs. Utilizing capital market data and custom algorithms, we evaluated the relative financial performance, including capital gains and total market returns, of various grocery retailers. Our analysis involved benchmarking their performance against the overall stock market for the period spanning from 2017 to 2022.

Starting with the paradox perspective framework on corporate sustainability (10), we extended the model to include a historic perspective, such that our findings are organized using Canadian grocers’ historical, descriptive, instrumental, and normative aspects. Our analysis focused on the social and financial outcomes of Canadian grocers.

Through data collected from consumers, corporate watchdogs, and retailers, we analyzed the moral landscape of Canadian grocers to understand how stakeholders experienced the impact of increased food inflation.

## Findings

**Figure 1:**
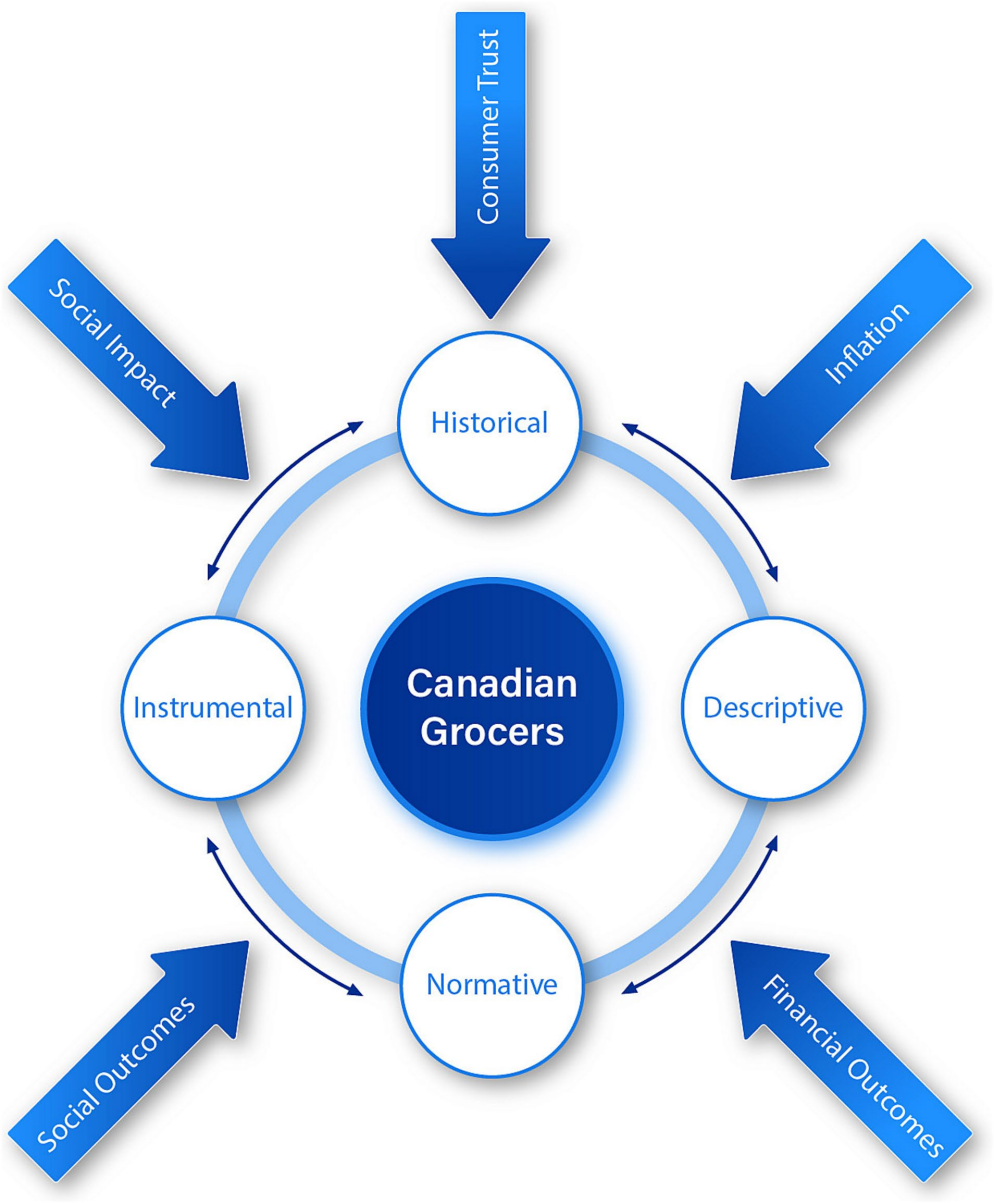
Analyzing the moral landscape of Canadian grocers, a conceptual model.

We extend model of Hahn et al. (10) to include a historical aspect to analyze the paradox perspective framework of corporate sustainability, as outlined in our conceptual model in Figure 1. As such, our findings are organized according to the aspects in this model: historical, descriptive, instrumental, and normative.

### Historical

The history of the Canadian grocery industry is marked by a story of consolidation. The prominent players in today’s landscape were not always the expansive and dominant entities they are now, nor did they always possess the substantial market share they currently hold. The origins of Canada’s modern grocery giants would appear quite unfamiliar to today’s consumers. In this article, we provide a concise historical overview of the industry, organized as follows: we begin with an overview of key historical facts, followed by an examination of the market dynamics and regulatory pressures that led to the market entries and mergers. We conclude by drawing a comparative analysis with the grocery industries in the United States and the United Kingdom.

#### Historical facts

We can briefly summarize the history of Canada’s major grocers to gain insight into their current market positions. Loblaw and Sobeys originated as limited-offering grocers around the conclusion of the First World War and underwent substantial expansion following the Second World War.

Loblaw began with a cash and carry model for grocery in 1919, adding todays well known staples such as meat and produce in 1933 (47). Similarly, Sobeys began offering a full line of produce to customers in 1924 (48). In 1947, a handful of grocery buyers joined together as Lasalle Stores Ltd., (later becoming Metro in 1972) (49).

Loblaw was incorporated in 1956, joining stores in Ontario and the United States into a single entity (50). In 1961, Giant Tiger is founded (51). Purchases then continue to fuel moderate expansion by the three major grocers, but major mergers, market entries, and acquisitions are rare between 1960 and 1980. In 1985, Costco joined the Canadian Market, opening its first store in British Columbia (52). Walmart subsequently entered Canada in 1994 with its purchase of Woolco Stores in Canada (53).

By the mid-1990s, the major grocers’ operations are highly resemblant of their current day businesses. What remains are the major acquisitions of the 1990, 2000, and 2010s. 1998 was a significant year for the industry in Canada. It was then when Sobeys purchased Oshawa Group (which included most IGA stores), and when Loblaw purchased Provigo (19). With well-established market positions, the grocers intensified their diversification of product offerings in regard to food [for example, the (53) purchase of T&T, an Asian grocery chain (54)].

Very recent merger and acquisition activity has only increased the degree of consolidation, with high profile acquisitions such as the purchase of Safeway by Sobeys Inc. (21), and Shoppers Drug Mart by Loblaw (in 2014) (55). Metro completed its own drugstore acquisition in 2018, buying Jean Coutu (56) while Sobeys completed its acquisitions of Farm Boy in 2018 (48), and Longos in 2021 (57).

By 2017, five companies (Loblaw, Sobeys, Metro, Walmart, and Giant Tiger) held over 70% of the Canadian grocery market (58). In the same year, there were approximately 7,000 independent grocery and convenience retailers remaining. By 2018, Costco and Walmart held market shares of 11 and 8%, respectively (59). At this point, the 7,000 independent grocery and convenience stores held approximately 20% of the market share (59).

#### Pressures leading to consolidation

In examining the factors that have contributed to the present state of the Canadian grocery industry, it may be beneficial to focus on the more recent history, starting in 1998. This marks a significant point in time, as it coincided with the entry of major American firms such as Costco and Walmart into the Canadian market. These market entries can be seen as a defining moment that separates the past from the current state of the Canadian grocery industry. Prior to 1998, market consolidation was primarily driven by the pursuit of greater scale, integration, and product diversity.

##### Market pressures

“The entry of United States competitors appears to have had a significant impact on market pressures, with Canadian grocers now focusing on competition with United States entrants. This implies that American firms can leverage their larger scale to pass on price efficiencies to their customers, necessitating an increased scale for Canadian grocers to remain competitive. Regarding the Empire-Oshawa deal, George Fleishmann, then President of the Food and Consumer Product Manufacturers of Canada, was quoted as saying, “The consolidation is necessary as the Canadian industry prepares itself for competition from larger North American discount operations such as Costco and Walmart’s new venture into the grocery business” (60).

Of the Provigo-Loblaw deal, the then commissioner of the Competition Bureau concluded “that increased competition is anticipated within the market from warehouse clubs, such as Costco, or mass merchandisers, such as Wal-Mart” (61). Later mergers do, however, still rely on arguments about promoting price efficiencies through synergies and a need to increase diversity in product offering. For example, Loblaw had the following to say about the acquisition of Shoppers Drug Mart: “It strengthens both companies’ competitiveness in an evolving retail landscape, creating new growth opportunities for shareholders. It will give consumers more choice, value and convenience” (62).

##### Regulatory pressures

The regulatory pressures involved in the industry’s dealmaking can be best characterized by a seemingly paradoxical lack of pressure. As previously mentioned, when discussing the Provigo-Loblaw deal, the Commissioner of the Competition Bureau pointed out that increased competition was expected from retail giants like Costco and Walmart. In the case of the Loblaw-Provigo deal, the Competition Bureau mandated that Loblaw divest itself of 18 stores and nine pharmacy operations (63), despite gaining more than 1,200 drug stores across all provinces from the deal (20). In the Sobeys-Oshawa deal the Competition Bureau required Sobeys to divest of five stores and a distribution center, although the company had over 700 stores already at the time (64). The small-scale divestitures mandated by the Competition Bureau underscore the Bureau’s regional approach to competition analysis. This approach seems aimed at preventing specific regions from being devoid of competitive grocery options within predetermined areas. Nevertheless, there appears to be a lack of substantial evidence regarding the analysis of these divestitures’ potential impact on the overall grocery market in Canada.

#### Comparison to other countries

When examining the history of Canadian grocery retail, it is pertinent to inquire whether Canada exhibits any unique characteristics when compared to its peer nations. The United States stands as a suitable point of comparison both in terms of geography and culture, while the United Kingdom provides a valuable perspective in terms of culture and politics. It is worth noting that the Overton Window in the United Kingdom may be positioned in a manner similar to that of Canada. Turning our attention to the United States, we observe a comparable level of market concentration among the top five grocery retailers. In 2017, these top five retailers collectively held a 66% market share, though it is important to highlight that certain regions exhibited even greater concentration. For instance, in the southern region of Texas, a striking 87% of the market share was controlled by just two companies (58).

In the United Kingdom, there exists a notable level of market concentration among the major industry players, although this concentration is comparatively lower than that observed in Canada and the United States. According to a 2021, MarketLine report focusing on the Food and Grocery Retail industry in the United Kingdom, “The market is primarily controlled by prominent multinational corporations.” The four most significant retailers in this sector—Tesco, Sainsbury’s, Asda, and Morrisons—collectively command more than half of the market share (65). Although these countries exhibit a high degree of concentration among top players, Canada stands out as unique in that its degree of concentration is even higher.

Considering that Canada has reached a juncture where the three largest Canadian public grocery retailers exert significant control over the market, our analysis will concentrate on these three companies. We will present our findings pertaining to Canadian grocers by employing the three facets of the paradox perspective on corporate sustainability, namely the descriptive, instrumental, and normative aspects.

### Descriptive

We have looked into media, including coverage on operating segment results, as well as corporate press releases and published interviews that describe and explain the moral landscape of Canadian grocers.

#### Media

Canadian grocers have garnered significant media attention in recent years due to issues such as bread price-fixing, labor union conflicts, the discontinuation of COVID-19 hero pay, and the emergence of record-high food prices and profits.

The Competition Bureau’s ongoing investigation into alleged price-fixing aimed at artificially inflating bread prices was publicly disclosed in 2017. This disclosure subsequently led to a class-action lawsuit being filed on behalf of Canadian consumers (66). Loblaw Companies Ltd., along with several of its subsidiaries, and George Weston Ltd. were the sole entities offering compensation in the form of $25 gift cards to customers (66). Other entities mentioned in the lawsuit, such as Metro Inc. and Sobeys Inc., have denied any involvement (66).

Grocery prices and corporate profits have soared over the last year, while grocery store workers continue to battle grocery giants in the quest for fair treatment. A recent example can be seen when more than 500 workers were laid off at a Loblaw distribution center in Calgary in 2022. Many workers are on strike for fair wages with Loblaw only offering a “straight money offer” with no regulations around working conditions (67).

“Hero pay” was a term employed to describe the additional compensation provided to front-line workers, including grocery retail staff, whose efforts enabled Canadians to access essential goods and services during the COVID-19 lockdowns. This pay was introduced in March 2020 by several grocery chains and was discontinued in the first half of June of the same year, sparking controversy (68).

It is not unexpected that grocery retailers are currently under heightened scrutiny due to the growth in both gross and net margins in absolute dollar terms. This scrutiny comes at a time when many Canadian families are grappling with decisions concerning the preservation of discretionary income or even how to provide for their households. In contrast, the concept of shareholder primacy posits that the primary purpose of a corporation is to generate returns for its shareholders, with decision-making oriented toward a singular objective: maximizing shareholder value (69).

In the mainstream media, plenty of attention has been focused on high food prices, which are an important driver of CPI. They also hit lower-income households the hardest. Several industries engage in the sale and production of food: grocery stores, the manufacturers of food and soft drinks, alcohol, tobacco and cannabis, as well as the agriculture industry itself. Three of these four industries have had higher pre-tax profits in 2021 than before the pandemic (70).

Food and beverage stores, such as grocery stores, were the biggest winners in this segment. They made $3.9 billion more pre-tax profit in 2021 than they did before the pandemic. Grocery stores faced higher input costs, as their cost of goods hit a record high in the third quarter of 2021. But these stores could pass that entire cost increase on to consumers while adding price markups. The industry hit record-high profit margins in the first quarter of 2021, squeezing more profit out of every dollar that it received in revenue compared to any other time in history (70).

On the other hand, the agriculture sector (i.e., Farmers) saw its profits cut in half in 2021 compared to pre-pandemic times, largely due to the drought in the summer of 2021. All other food industries that are higher up in the supply chain hit new all-time highs in profits and/or profit margins while farmers literally felt the heat (70).

##### Operating segments

Required reporting for operating segments for public companies is laid out by International Financial Reporting Standard 8 Operating Segments. “Two or more operating segments may be aggregated into a single operating segment if aggregation is consistent with the core principle of this IFRS, the segments have similar economic characteristics, and the segments are similar.” Annual reports for Canadian grocery retailers report all grocery results as one segment on the premise that the nature of the products and services are similar.

The Kroger Co.’s Notice of 2022 Annual Meeting of Shareholders 2022 Proxy Statement and 2021 Annual Report on Form 10-K states: “The Company’s retail operations, which represent 97% of the Company’s consolidated sales, are its only reportable segment” (23).

Metro indicates in their Q3 interim financial report, “We are pleased with the performance of our food and pharmacy businesses in the third quarter, which was achieved in a challenging operating environment with increasing inflationary pressures” (71).

“The thing is, Canada’s major grocers, a group that also includes Metro Inc., have the ability to help lay the charges to rest. They decline to do so, however, using a loophole in accounting standards to obscure the facts about their operations. Their decisions to not publish greater detail on their businesses deprives the investing public of information that allows it to better evaluate the companies’ shares, and the general public of information that might help address the policy question of whether grocers have indeed ‘profited’ from inflation” (72).

Loblaw and its peers are failing at that objective. For the sake of shareholders and Canadians, it is time to get more disclosure about the parts of grocers’ businesses that are driving expanding profit margins (72).

#### Press releases and published interviews

Reactions from the major players in the grocery industry can be characterized as indignant, citing a lack of understanding of the underpinnings of the business and compliance with requisite accounting regulations.

Asked for additional comment, Loblaw spokesperson Catherine Thomas said: “We’re comfortable with our continuous disclosure. It meets all accounting and legal requirements and provides our investors with an appropriate amount of color on our business.” Metro declined to comment beyond its disclosures, while Empire did not respond to The Globe’s queries (72).

In a letter shared with some of its customers on Monday, Loblaw chairperson and president Galen G. Weston says the price of an average basket of groceries is up about 10 per cent this year, with such items as apples, soup, and chips up even more. Weston said much of this is “maddeningly” out of the company’s control as food suppliers pass on higher costs to Loblaw (73).

### Instrumental

The instrumental aspect looks at short and long-term outcomes. Short term outcomes tend to be less than 1 year and are displayed as aggregated interim data for recent quarterly performance. Long-term outcomes of public market grocers may be measured with financial returns over a period of years.

#### Interim data

The financial analysis of grocery quarterly earnings for the calendar year 2022 is shared with permission from Dalhousie University’s Agri-food Analytics Lab (3).

We found the best and average gross profit for the most recent 5 years were 25.47 and 24.88% for Empire/Sobeys,19.99 and 19.82% for Metro, and 31.47 and 30.45% for Loblaw. We compared this historical performance against each company’s most recent two quarters, and our findings are in Table 1 (3).

**Table 1:** Canadian grocer historical performance.

	Empire/Sobeys		Metro		Loblaw	
CAD in Millions	22-Apr	22-Jul	22-Mar	22-Jun	22-Mar	22-Jun
Revenue	$7,840.8	$7,937.6	$4,274.2	$5,865.5	$12,262.0	$12,847.0
Cost of revenue	$5,836.8	$5,959.7	$3,416.8	$4,706.8	$8,335.0	$8,692.0
Gross profit	$2,004.0	$1,977.9	$857.4	$1,158.7	$3,927.0	$4,155.0
Gross profit % − Actual	25.56%	24.92%	20.06%	19.75%	32.03%	32.34%
Gross profit % − 5-year average threshold	24.88%	24.88%	19.82%	19.82%	30.45%	30.45%
Gross profit in excess of 5-year average threshold (%)	0.68%	0.04%	0.24%	−0.07%	1.58%	1.89%
Gross profit in excess of 5-year average threshold ($)	$53.2	$3.0	$10.3	-$3.8	$193.2	$243.1
Gross profit % − 5-year best threshold	25.47%	25.47%	19.99%	19.99%	31.47%	31.47%
Gross profit in excess of 5-year best threshold (%)	0.09%	−0.55%	0.07%	−0.24%	0.56%	0.87%
Gross profit in excess of 5-year best threshold ($)	$6.9	-$43.8	$3.0	-$13.8	$68.1	$112.0

All large grocers have an increase in gross profit in 2022 relative to their average past performance: Empire/Sobeys earned an excess of $56 million; Metro earned an excess of $7 million, and Loblaw earned an excess of $436 million. We observed Empire/Sobeys’ performance in 2022 was overperforming relative to their best years in Q2 by $7 million, while their Q3 numbers had them underperforming by $44 million. For the most recent two quarters of 2022, Empire/Sobeys had a net deficit of $37 million relative to their best years’ performance (3).

When looking at performance of Metro (49) relative to its best years, we noted they were overperforming in Q1 by $3 million and underperforming by $14 million in Q2. For the most recent two quarters of 2022, Metro Inc. had a net deficit of $11 million relative to their best years’ performance. In Q1 2022, Loblaw outperformed their best years’ performance equivalent to $68 million; in Q2 2022, they outperformed their best years’ performance by $112 million. In total, Loblaws’ gross profit thus far in 2022 outperforms its best performance of the past 5 years by $180 million (3).

#### Corporate returns

Corporate returns reflect the market’s assessment of the financial strength and viability of a corporation. We have included both the capital gains (i.e., growth in share price) and the total market returns (i.e., capital gains and dividends) to compare the results of each public company grocers’ strategic results.

##### Capital gains

**Figure 2:**
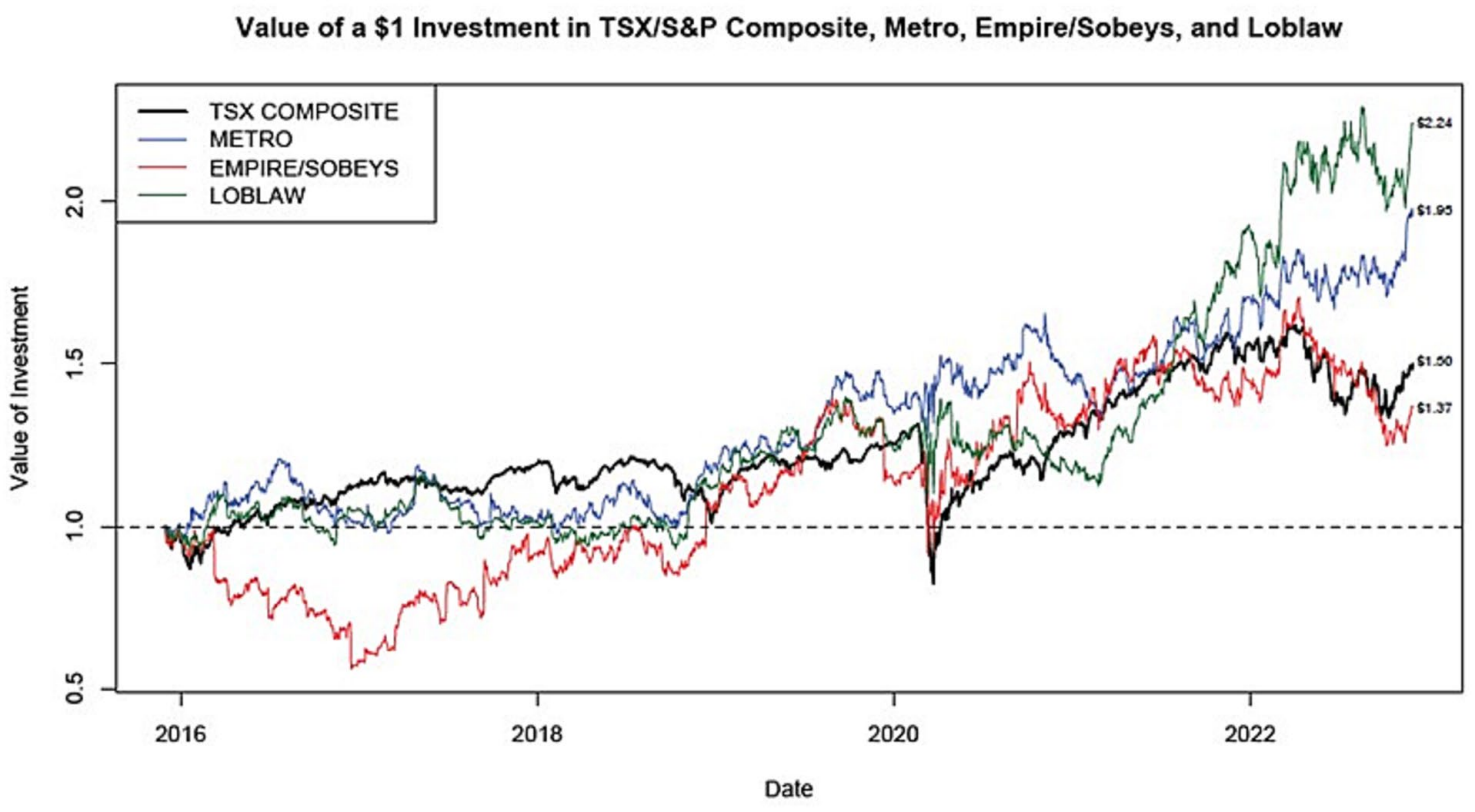
Market returns for Canadian grocers 2016–2022.

Capital gains returns reflect the market’s assessment of the strength and viability of a corporation as perceived by current and potential investors. Figure 2 shows the market returns for Canadian grocers from 2016 to 2022.

For a period of 5 years (December 1, 2017, to November 30, 2022) a $1 investment in each of the Canadian public grocers grew to $2.24 (Loblaw), $1.95 (Metro), and $1.37 (Empire/Sobeys). Over the same period, $1 invested in the TSX/S&P Composite would have grown to $1.50.

##### Total market

**Figure 3:**
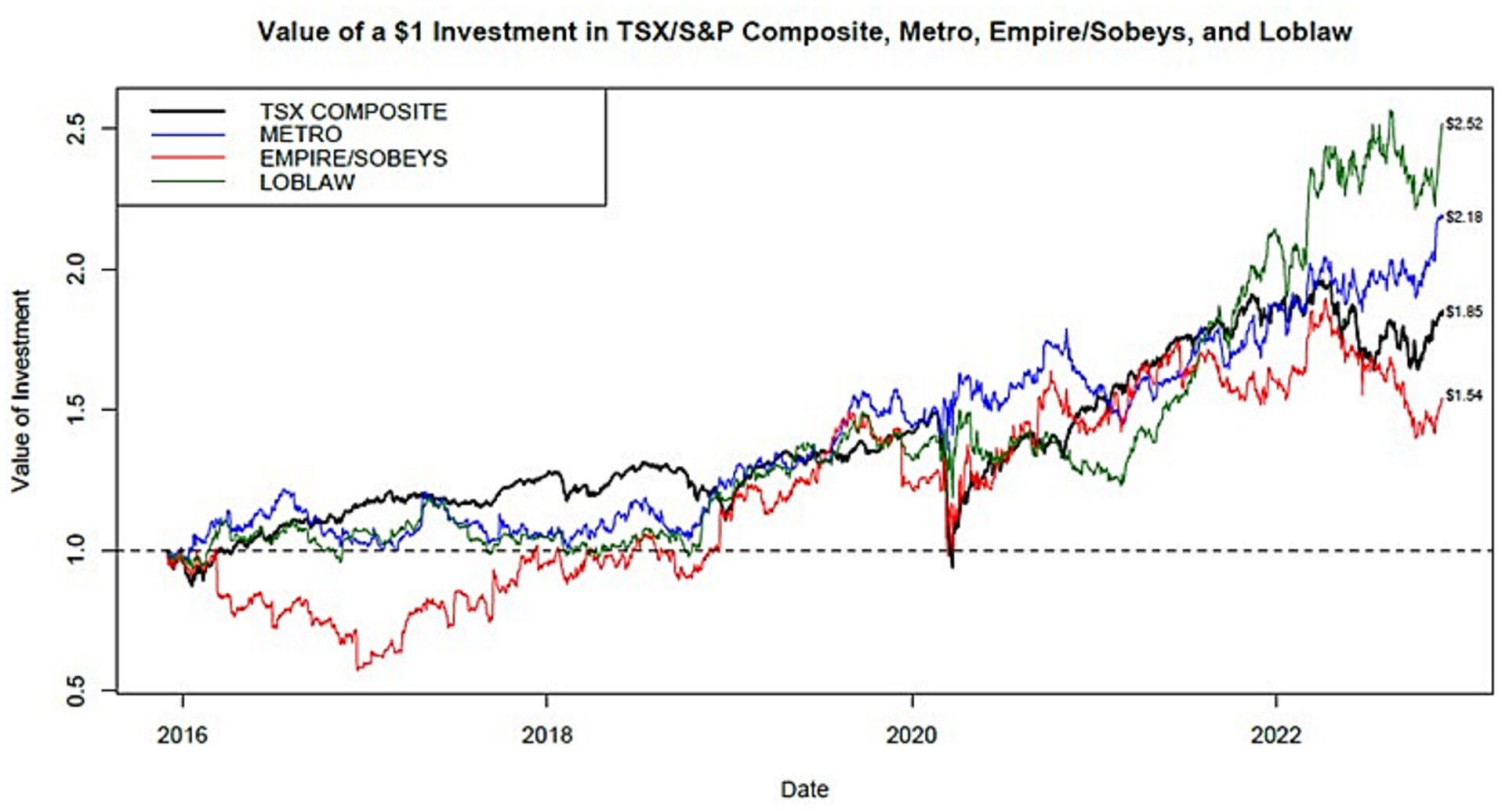
Total market returns for Canadian grocers 2016–2022.

Total market returns reflect the market’s assessment of the strength and viability of a corporation as perceived by current and potential investors and the dividends declared and paid by the corporation. Figure 3 shows the total market returns for Canadian grocers 2016–2022.

For a period of 5 years (December 1, 2017, to November 30, 2022) a $1 investment in each of the Canadian public grocers provided a total return of $2.52 (Loblaw), $2.18 (Metro), and $1.54 (Empire/Sobeys). Over the same period, $1 invested in the TSX/S&P Composite would have returned $1.85.

### Normative

The normative aspect allows for corporations to be a part of a greater discussion of the role of sustainable business practices. Given the lack of regulation of food prices in the Canadian mixed-market economy, it follows the lack of current normative practices of Canadian grocers.

#### Competition bureau investigation

On October 24, 2022, Canada’s Competition Bureau announced they would be investigating the Canadian food supply chain. Below are the main questions addressed in that press release (74).

The study will examine three main questions: To what extent are higher grocery prices a result of changing competitive dynamics? What can we learn from steps that other countries have taken to increase competition in the sector? How can governments lower barriers to entry and expansion to stimulate competition for consumers?

## Discussion

Our findings suggest that record food inflation and a lack of transparency have eroded consumer trust in grocers. At a time of unprecedented inflation, a dearth of regulations, and increased consumer ability to voice concerns, a storm of “Greedflation” anxieties has emerged. A Canadian grocer has been found guilty of price-fixing for bread and may have engaged in tacit collusion to eliminate “hero pay.” Some have even resorted to threatening union lockouts when workers requested improved working conditions. Simultaneously, grocers are enjoying favorable financial and market returns.

Gross profit is the difference between the prices grocers charge (revenues) and the cost of putting items on their shelves (cost of revenues). When discussing “Greedflation,” the concern is that grocers are capitalizing on high inflation to set prices unreasonably high. However, determining what constitutes “excessive” profit is challenging. By examining a company’s gross profit percentage (gross profit divided by sales multiplied by 100), we can establish a relative basis for year-over-year analysis. All three Canadian public grocers are outperforming the average in terms of gross profit, with the largest grocer, Loblaw, achieving its best gross profit performance yet (3).

To address their record-high performance, Loblaws earnings press release stated Q2 News Release attributes of Loblaw Reports 2022 Second Quarter Results (75) its increase in sales to an increase in same-store sales for food retail (0.9%) and drug retail (5.6%). This does not necessarily imply that the increase in gross profit can be attributed to the 0.9 and 5.6% respective increases in revenues from food and drug. However, the manner in which the information is presented may give such an impression. Similarly, when examining Metro’s Q3 financial statements concerning their food and pharmacy businesses, it appears that the retailer also treats these as distinct segments. This raises questions about how grocery retailers interpret what qualifies as a reportable segment. If annual reports provided a more detailed breakdown of results, the public would have a better opportunity to gain a clearer understanding of the underlying dynamics.

Due to current international reporting standards, Loblaws is not required to disclose the gross profit for each operating line (e.g., food, drugs, clothing, and beauty). Under international reporting standards, companies may aggregate operating segments with economically similar characteristics when segments are similar in all respects of the nature of, and customers for, their products and services, production, methods of distribution, and nature of the regulatory environment (76). IFRS does not provide a fulsome definition of the term “economic characteristics.” However, they do provide an example within the text of IFRS 8 in section 12 of the standard which reads:

Operating segments often exhibit similar long-term financial performance if they have similar economic characteristics. For example, similar long-term average gross margins for two operating segments would be expected if their economic characteristics were similar.

While this is a useful starting point for understanding what IFRS means when using the term “economic characteristics,” the picture becomes clearer when combined with the core principle of the standard. Section 1 reads:

An entity shall disclose information to enable users of its financial statements to evaluate the nature and financial effects of the business activities in which it engages and the economic environments in which it operates.

These two sections, in combination, allow us to make a reasonable inference about the spirit of the standard. In our view, IFRS’ intention is for reporting entities to disclose separate segments that have materially different gross margins because a financial statement user making an investment decision would care about those margins. A conjured example helps illustrate the point: If an investor were trying to decide whether or not to invest in Loblaw but found out that they have a margin of −10% on food, and + 30% on drugstore and clothing, they might believe it prudent to use their input as a shareholder to encourage the company to divest of its food business, and simply remain a drugstore and clothing store.

Under the current situation, it is impossible for users of financial statements to make this determination, which we would say is material to the investment decision making process. As recently as July 2022 Loblaw had told the public that their increased profits were due to their drugstore products, and not grocery products (77). This could be due to changes in volume of sales but also different gross margins being provided by these assorted products. Hence, we would say that they are not reporting within the spirit of the standard that is IFRS 8.

Canadian public grocery retailers are subject to quarterly reviews and annual audits as a requirement of their public company status in Canada. Consequently, management assertions and judgments regarding operating segments have undergone evaluation by third-party auditors and have been found to be free of material misstatements. This implies that although it may not be immediately evident that the grocery industry shares similarities in terms of its nature, customer base, or production processes with other sectors such as pharmaceuticals, clothing, or beauty, both grocery store management and auditors have established a sufficient basis to conclude that they are, in fact, similar (to a reasonable extent).

Nevertheless, it is important to note that any changes in food-related regulations in Canada could potentially impact the interpretation of the last part of IFRS 8.12, specifically the section addressing the ‘nature of the regulatory environment.

Food is a fundamental human necessity, akin to housing, heating, and transportation. Unlike food prices, which are subject to market forces, housing, utilities, and specific segments of the transportation sector in Canada are influenced by regulatory measures aimed at ensuring equitable pricing for Canadians. In cases where there is a dearth of competition, such as in certain provincial utilities, these industries are subject to comprehensive government oversight. Nearly three-quarters of Canadians contend that they face challenges in satisfying their essential needs (78). Simultaneously, Canadian grocers continue to experience consistently robust economic performance, showing no signs of decline. This prompts consideration for enhanced government oversight regarding the pricing of food products for Canadian consumers.

Government regulation of food retailers could play a pivotal role in ensuring Canadians can adequately provide for their families while fostering increased transparency in the reporting of results within Canadian grocery store operating segments. Should regulations be introduced for grocery financial reporting, it is plausible that grocer management and third-party auditors may face heightened challenges when attempting to consolidate sales and gross margins from distinct categories such as food, drugs, clothing, and beauty.

Recent press releases from grocers have seen the release of select pieces of information, often used to justify their heightened gross profit margins, with claims that food sales have remained “relatively flat,” while gross profit margins on pharmaceutical drugs have witnessed an increase. Remarkably, the public response does not seem to express outrage toward grocers making increased profits on pharmaceuticals. This observation suggests that if grocers indeed experience heightened gross profits in other lines of business, increased transparency in reporting could serve to earn consumers’ trust, consequently reducing the scrutiny placed on corporate profits. In other words, the perception of corporate profiteering in the food sector may be ameliorated through enhanced transparency in operating segments, which distinctly delineate gross profits from food and other product lines.

Our research findings strongly indicate that record food inflation and the absence of transparency have adversely impacted consumer trust in grocers. However, the extent to which this has influenced consumer purchasing behavior remains unclear. Future research endeavors could delve into the intricate relationship between consumer trust and food inflation, shedding light on the potential consequences for consumer behavior in the grocery retail sector.

## Conclusion

Forty-year record-high inflation and unprecedented food prices are currently impacting Canadians. Consequently, consumers are calling for increased government oversight. The implementation of a mandatory grocer code of conduct and enhanced transparency in financial reporting operating segments would empower stakeholders to assess the sources of grocer profits independently. Through these measures, grocers may begin to rebuild the trust that has been eroded among consumers. Paradoxically, when social needs are no longer perceived as neglected or exploited, corporate profits may indeed continue to rise, but this time with reduced scrutiny and social unrest.

## Data availability statement

The raw data supporting the conclusions of this article will be made available by the authors, upon reasonable request.

## Author contributions

ST: Conceptualization, Data curation, Formal analysis, Investigation, Methodology, Project administration, Resources, Writing – original draft, Writing – review & editing. SC: Conceptualization, Data curation, Formal analysis, Investigation, Methodology, Resources, Validation, Writing – review & editing, Writing – original draft. TC: Data curation, Formal analysis, Investigation, Writing – original draft, Writing – review & editing. BC: Data curation, Formal analysis, Investigation, Software, Validation, Visualization, Writing – original draft, Writing – review & editing.

## Footnotes

^1^“In September, prices for food purchased from stores (+11.4%) grew at the fastest pace year-over-year since August 1981 (+11.9%). Prices for food purchased from stores have been increasing at a faster rate than the all-items CPI for 10 consecutive months, since December 2021” (2).
